# Measuring prefrontal cortical activity during dual task walking in patients with Parkinson’s disease: feasibility of using a new portable fNIRS device

**DOI:** 10.1186/s40814-016-0099-2

**Published:** 2016-09-23

**Authors:** Freek Nieuwhof, Miriam F. Reelick, Inbal Maidan, Anat Mirelman, Jeffrey M. Hausdorff, Marcel G.M. Olde Rikkert, Bastiaan R. Bloem, Makii Muthalib, Jurgen A.H.R. Claassen

**Affiliations:** 1 Donders Institute for Brain, Cognition and Behaviour, Nijmegen, The Netherlands; 2 Departments of Neurology, Geriatric Medicine, and Radboud Alzheimer Center, Radboud University Medical Center, Nijmegen, The Netherlands; 3 Department of Neurology, Center for the study of Movement, Cognition, and Mobility, Tel Aviv Sourasky Medical Center, Tel Aviv, Israel; 4 Sackler Faculty of Medicine, Tel Aviv University, Tel Aviv, Israel; 5 EuroMov, University of Montpellier, Montpellier, France

**Keywords:** Spectroscopy, Near-infrared, Gait, Parkinson’s disease, Prefrontal cortex, Hemodynamics

## Abstract

**Background:**

Many patients with Parkinson’s disease (PD) have difficulties in performing a second task during walking (i.e., dual task walking). Functional near-infrared spectroscopy (fNIRS) is a promising approach to study the presumed contribution of dysfunction within the prefrontal cortex (PFC) to such difficulties. In this pilot study, we examined the feasibility of using a new portable and wireless fNIRS device to measure PFC activity during different dual task walking protocols in PD. Specifically, we tested whether PD patients were able to perform the protocol and whether we were able to measure the typical fNIRS signal of neuronal activity.

**Methods:**

We included 14 PD patients (age 71.2 ± 5.4 years, Hoehn and Yahr stage II/III). The protocol consisted of five repetitions of three conditions: walking while (i) counting forwards, (ii) serially subtracting, and (iii) reciting digit spans. Ability to complete this protocol, perceived exertion, burden of the fNIRS devices, and concentrations of oxygenated (O_2_Hb) and deoxygenated (HHb) hemoglobin from the left and right PFC were measured.

**Results:**

Two participants were unable to complete the protocol due to fatigue and mobility safety concerns. The remaining 12 participants experienced no burden from the two fNIRS devices and completed the protocol with ease. Bilateral PFC O_2_Hb concentrations increased during walking while serially subtracting (left PFC 0.46 μmol/L, 95 % confidence interval (CI) 0.12–0.81, right PFC 0.49 μmol/L, 95 % CI 0.14–0.84) and reciting digit spans (left PFC 0.36 μmol/L, 95 % CI 0.03–0.70, right PFC 0.44 μmol/L, 95 % CI 0.09–0.78) when compared to rest. HHb concentrations did not differ between the walking tasks and rest.

**Conclusions:**

These findings suggest that a new wireless fNIRS device is a feasible measure of PFC activity in PD during dual task walking. Future studies should reduce the level of noise and inter-individual variability to enable measuring differences in PFC activity between different dual walking conditions and across health states.

**Electronic supplementary material:**

The online version of this article (doi:10.1186/s40814-016-0099-2) contains supplementary material, which is available to authorized users.

## Background

Many patients with Parkinson’s disease (PD) have difficulties to perform a second task while walking (i.e., dual task walking), such as walking and talking or walking while paying attention to passing traffic [1]. As a consequence of performing two tasks at the same time, gait and/or their performance on the secondary task at hand deteriorates [2–4]. These difficulties in dual task walking often lead to increased disability, increased fall risk, and reduced quality of life [1, 5, 6].

Mechanisms underlying difficulties in dual task walking are largely unclear. However, the prefrontal cortex (PFC), which is involved in human balance and locomotion [7], likely plays an important role. Although cognitive functions depending on the PFC are often affected in PD [8–10], patients may rely more on the PFC due to reduced movement automaticity of dysfunctional basal ganglia circuits [1, 11–13]. Therefore, altered functioning of the PFC during dual task walking in PD might explain their difficulties and should therefore be further examined.

Functional near-infrared spectroscopy (fNIRS) is a promising method for measuring PFC activity during dual task walking [14–16]. With fNIRS, relative concentrations of oxygenated (O_2_Hb) and deoxygenated (HHb) hemoglobin can be measured [17, 18]. In typical neural activity as measured with fNIRS, increases in O_2_Hb and stable or slight decreases in HHb are present [14, 16, 18, 19]. The use of fNIRS offers several advantages over other neuroimaging techniques. Compared to functional magnetic resonance imaging (fMRI), which has a higher spatial resolution and can reach subcortical areas, fNIRS is lightweight, easy to use, low cost, and can be portable [11, 14, 18]. Compared to electroencephalography (EEG), which can also be used during actual walking [20], fNIRS can provide higher spatial resolution, is easier to use, and more robust to head movement [14, 18, 21]. These advantages make fNIRS particularly attractive to be used for measurement during actual walking [11].

In healthy young and elderly persons, fNIRS was used to detect increased PFC activity during walking while talking [22, 23], while counting [24], and while serially subtracting [24–26]. Compared to these populations, PD patients generally are physically less fit. This might limit their ability to perform multiple task repetitions, which are needed for reliable fNIRS measurements. In addition, PD patients often show altered movement patterns such as restricted gait, dyskinesia, or bradykinesia. This might influence head position and head movement during walking and thereby further restrict the feasibility of using fNIRS to measure PFC activity during dual task walking.

Therefore, the primary aim of this pilot study was to examine the feasibility of measuring bilateral PFC activity in patients with PD during different dual task walking conditions using two lightweight, wireless fNIRS devices (Portalite fNIRS system). This was done in the context of the V-TIME project [27, 28] in which we applied fNIRS to study the role of the PFC in complex walking in PD patients. The first results of this project, which were recently published [13], focus on potential mechanisms underlying dual task difficulties in PD. Although outside the scope of this recently published work, details of pilot work concerning methodology and feasibility of using fNIRS in PD are crucial for the development of new studies to further disentangle the neural mechanisms underlying dual tasking in PD.

As a start for the development of appropriate protocols for such studies, this feasibility study incorporated several specific goals. First, we assessed feasibility by testing whether PD patients were able to perform several repetitions of dual task walking while wearing the devices and by registering their experience and perceived exertion. Second, we aimed to investigate whether we were able to record the expected typical fNIRS signal of neuronal activity in the PFC (the neuronal hemodynamic response [18]) as a consequence of dual task walking when compared to rest. We tested this at a group level, and at individual level, to explore potential inter-individual variability. Finally, we explored the sensitivity of our method to detect differences in O_2_Hb and HHb concentrations between dual task walking and rest and between dual tasks.

## Methods

### Participants

We recruited PD patients (*N* = 14) from the outpatient clinic of the Neurology Department of the Radboud University Medical Center in the Netherlands. This number of 14 participants was based on similar studies in other populations, in which 6 [29], 11 [22], and 17 [25] participants were sufficient to measure PFC activity during walking tasks. Inclusion criteria were (1) age 60–85 years, (2) clinical diagnosis of idiopathic Parkinson’s Disease, according to the UK Brain Bank criteria, (3) Hoehn and Yahr stage II to III (while on medication), (4) an increased risk of falling as indicated by the treating physician, or reflected by actual falling incidents within 6 months prior to the study, and (5) able to walk at least 5 min without help (walking aids were allowed). Exclusion criteria were (1) psychiatric co-morbidities, (2) neurologic co-morbidities in medical history, (3) co-morbidity of the motor system which restricts gait, (4) clinical diagnosis of dementia, (5) unable to comply with the test protocol, and (6) severe freezing precluding safe participation.

### Baseline characteristics

General demographic characteristics were assessed by questionnaires. Education level was assessed based on the Dutch education system [30] using seven categories (1 = less than primary school, 7 = university degree). The Longitudinal Aging Study Amsterdam Physical Activity Questionnaire (LAPAQ) [31] was completed to obtain physical activity levels of the participants in 2 weeks before assessment, the Mini-Mental State Examination (MMSE) to assess global cognitive functioning [32], and the Fall Efficacy Scale International (FES-I) [33] to assess fear of falling. Fall rate, disease duration, and medication use were assessed by medical history taking of the subjects and proxies, with a fall being defined as “an unexpected event in which the participant comes to rest on the ground, floor or lower level,” which is consistent with the recommendations of the Prevention of Falls Network Europe (ProFaNE).

### Protocol/procedure

Participants were instructed to walk back and forth over a course of approximately 8 m which was marked by a cone at each end and to make wide turns around the cones. During all walking tasks, participants walked at their preferred pace in a quiet room with comfortable footwear. Participants were allowed to use a customary cane, but walkers were not allowed during assessments. Medication was used as normal, and thus, all tests were performed in the on-medication state.

For dual task walking, three different types of tasks were used: walking while (1) counting forward, (2) serially subtracting, and (3) reciting digit spans. These tasks were chosen because it can reasonably be assumed that PFC activity is present during these tasks in PD [2–4, 34–39]. In previous studies, dual task effects were seen on behavioral outcome measures in PD during both walking while serially subtracting [2–4, 34–39] and while reciting digit spans [40]. In addition to these behavioral findings, fNIRS was successfully used in other populations to show increased PFC activity during walking while counting forward [24] and while serially subtracting [24–26].

During walking while counting forward, participants were asked to count forward at their own pace, starting from one. For serial subtraction, participants were instructed to continuously count backward alternating in steps of three or seven, starting from a number between 91 and 100. When a participant started with serial seven subtractions, we switched to serial three subtractions at the start of the next trial, or when zero was reached. The digit span consisted of repeating series of digits which the assessor said out loud. The number of digits to be repeated was based on the forward digit span of the Wechsler Adult Intelligence Scale (WAIS-III) [41]. Before starting any task, participants sat in a chair and repeated digit spans of increasing length. If subjects were unable to correctly repeat two out of three spans of the same length, the number of digits to be repeated during walking while reciting digit spans was one less than this length. If participants were unable to correctly repeat a span of this length during walking and stopped performing the digit span completely, the span length was reduced until participants did engage in the digit span during walking. This protocol was used to adjust the difficulty of reciting digit spans to each participant’s ability, thereby adjusting for baseline differences [40].

Each of the walking tasks was performed five times distributed over five blocks, with each block consisting of three different trials (one of every task). The order of these three trials within a block was randomized. Every trial started with 20 s of standing still, followed by 40 s of task performance, and another 20 s of standing still. During the two 20-s rest periods, participants were instructed to stand as quietly as possible, keep their heads still, look straight ahead, and think of nothing in particular. After 20 s of standing still, the assessor would say “start” for walking while counting, “start with [first number] minus [three or seven]” for walking while serially subtracting, and “start with [first digit span]” for walking while reciting digit spans. After this start sign, participants were instructed to start walking and simultaneously perform the cognitive task. After 40 s of task performance, the assessor said “stop,” after which the participant had to remain standing quietly where he or she ended for 20 s.

In between trials, participants walked back toward the start position and a rest period of random duration (1–2 min) was given in which instructions for the next trial were given. In between blocks, participants were allowed to sit down and rest until they were ready to continue. Before starting any trial, it was made sure the participant was standing for at least 1 min to minimize blood pressure fluctuations after standing up.

### Feasibility assessment

To judge the feasibility of the protocol, participants were asked to complete the Borg Rating of Perceived Exertion Scale (RPE) [42], ranging from 6 (very light effort) to 20 (very very hard) after each block. After completion of the protocol, participants were asked to complete a questionnaire about their experience. This questionnaire included: “Did the fNIRS system (Portalites) burden you while walking?” (5 point Likert scale: 1 = “No, not at all” and 5 = “Yes, a lot”) and “Was it doable to complete the protocol?” (5 point Likert scale: 1 = “Yes, very easily doable” and 5 = “No, undoable”).

### fNIRS system

In order to test whether we were able to measure the typical fNIRS signal of neuronal activity, we measured concentration changes in O_2_Hb and HHb in the PFC with the PortaLite™ fNIRS system (Artinis Medical Systems, Elst, the Netherlands). Like other fNIRS systems, this system uses near-infrared light which penetrates the skull and brain, but absorbed by hemoglobin (Hb) chromophores in the cortical layer microcirculation. Light was transmitted with two wavelengths, 760 and 850 nm, and data was sampled with a frequency of 10 Hz. The PortaLite™ uses wireless technology (Bluetooth), allowing participants to walk without restriction of wires. Two devices were placed on the forehead of the participants, one on the right and the other on the left side. Both devices were positioned at a height of 15 % of the nasion-inion distance from nasion, and we placed the middle of the device at 7 % of the head circumference to the left and right from midline, to avoid measuring the midline sinus. These locations roughly target left and right Brodmann’s areas 9 and 10, which represent the dorsolateral and anterior PFC [43, 44]. The devices were shielded from ambient light by covering the whole forehead with a black cloth. Oxysoft version 3.0.52 (Artinis Medical Systems, Elst, The Netherlands) was used for data collection.

Based on different Hb absorption spectra, concentration changes of O_2_Hb and HHb in the targeted PFC area were calculated from the changes in detected light intensity. This was done using the modified Lambert-Beer law, assuming constant light scattering [45]. A PortaLite™ has three transmitters and one receiver, with transmitter-receiver distances of 30, 35, and 40 mm. The differential path length factor (DPF), which accounts for the increased distance traveled by light due to scattering, was set to 6 for all participants. This is in line with previous studies in adults [46]. Although the DPF is age-dependent [46], no data is available on the actual DPF variation in adults older than 50. With the fixed DPF we chose, the assessment of relative changes in O_2_Hb and HHb within and between tasks will not be affected.

### fNIRS analysis

**Fig. 1:**
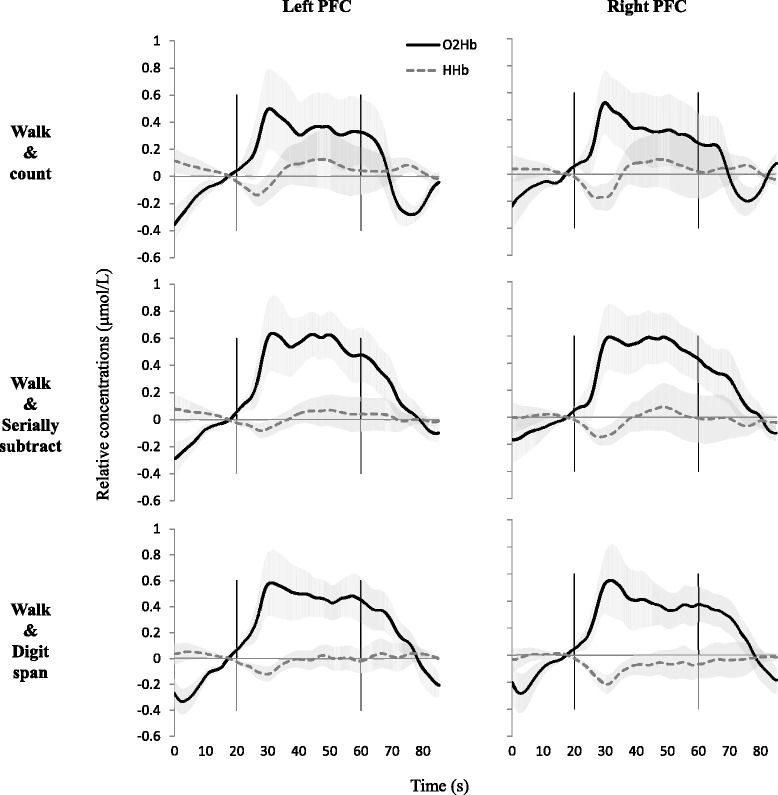
fNIRS signal time courses for each task. Average time courses of oxygenated hemoglobin (O_2_Hb: *dark*, *solid lines*) and deoxygenated hemoglobin (HHb: *lighter*, *dotted lines*) of all subjects (*N* = 12) for left and right prefrontal cortices, mean ± sem. *Vertical black lines* indicate start and end of task performance. *PFC* prefrontal cortex

Measured concentrations of O_2_Hb and HHb from each of the Portalite devices were exported to MATLAB (MATLAB and Statistics Toolbox Release 2012b, The MathWorks, Inc., Natick, Massachusetts, United States), in which further data processing was done. First, O_2_Hb and HHb signals of the three channels (the three transmitter—receiver distances) per PortaLite were averaged. Then, the moving standard deviation-based artifact removal (movement artifact reduction algorithm—MARA) method was performed within each trial [47]. The threshold for artifact detection was set to 0.45 for O_2_Hb and 0.18 for HHb, with a window length for moving standard deviation calculation at 0.5 s, and a window length for artifact correction (LOESS smoothing window) on 1 s. These parameters were chosen after testing several settings and visual inspection of the data by JC and FN on adequate removal of large (movement) artifacts while not affecting physiological fluctuations. Next, the fNIRS signals were linearly detrended per trial and low-pass filtered at 0.1 Hz using a Butterworth filter to remove heart rate and other higher frequency physiological signals. To enable direct comparison of the five different trials within each task, the filtered signals were biased, using the average concentration of the 5 s before the “Start” instruction as reference (zero). Then, individual trials were averaged per task to create three mean time course signals per person, which were then averaged over all participants (see Fig. 1). Finally, the mean concentrations (O_2_Hb and HHb) during the final 5 s of all 20-s rest periods were calculated over all trials for all participants and mean concentrations (O_2_Hb and HHb) of the 40 s after the “Start” instruction were calculated for each trial and then averaged for each of the three walking tasks.

### Gait performance analysis

To be able to judge whether our protocol was able to detect behavioral dual task effects and to interpret the level of PFC activity in relation to behavioral performance, gait parameters, and performance on the cognitive tasks were measured.

Walking performance parameters were measured on a GAITRite® mat (CIR Systems Inc., Clifton, NJ 07012, USA), over which the participants walked while walking forth over the 8-m course. The GAITRite® mat is an electronic roll-up walkway with pressure sensors embedded in a carpet. The carpet is 5.18-m long and 0.90-m wide, and the active area is 4.27-m long and 0.61-m wide. The mat is connected to a personal computer using GAITRite software version 4.0 (CIR Systems Inc., Clifton, NJ 07012, USA) for processing and storing the data. This system has been shown to be reliable and accurate for measuring walking parameters in elderly and in people with PD [48–50]. For investigating gait under dual task conditions in PD, it has been used as reference [51]. Automatic identification of footsteps was checked step by step for each trial and manually corrected where necessary. Walking performance parameters included gait speed, cadence, stride length, stride time, and the coefficient of variability (CV) of both stride length and stride time calculated as (standard deviation/mean)*100. Gait performance outcome measures were determined for individual trials first (with an average of 13.1 ± 2.9 footsteps) and then averaged over all trials within the three tasks for further analysis.

### Cognitive performance analysis

Cognitive performance measures included the number of subtractions and digit spans completed within the 40 s of task performance and the percentage of correct answers on both tasks. Outcome measures on gait and the cognitive tasks were determined for individual trials first and then averaged over all trials within the three tasks for further analysis.

### Statistical analysis

Statistical analysis was performed using IBM SPSS Statistics Version 21. To estimate the level of activity and noise, we calculated group means and 95 % confidence intervals (CI) for both O_2_Hb and HHb concentrations. Likewise, means and 95 % confidence intervals were calculated for the behavioral performance measures.

To explore differences between tasks in O_2_Hb and HHb concentrations and behavioral performance measures, we calculated Cohen’s *d*_z_ effect sizes based on mean difference scores [52]. Effect sizes of 0.2, 0.5, and 0.8 were interpreted as small, medium, and large, respectively [53]. Differences between dual walking tasks were tested for significance with the Wilcoxon signed-rank test, with *P* < .05 as threshold for statistical significance.

## Results

### Participants

After screening, 14 patients were found eligible and were included in the study. One of these patients was unable to safely perform the tests, and another patient came in tired and stopped after two blocks because of fatigue. These two participants (both male; 78 and 76 years old; 8 and 11 years since PD diagnosis; Hoehn and Yahr stage 2 and 2.5; 6 and 0 falls in the previous 6 months) were excluded from further analysis. Characteristics of the remaining 12 participants are shown in Table 1. For these participants, a total of 155 out of 180 intended trials were included in analyses. Reasons for dropped trials were inconsistent use of walking aid or inconsistent length of digit spans to be recited. No trials were dropped because of noisy fNIRS signals due to movement artifacts. For all participants, at least three trials per task were included in the analyses. Only one participant used a cane during the trials that were included in analyses.

**Table 1:** Characteristics of participants (*N* = 12)

Age (years)	70.1 ± *5.4*
Gender (men)	7
Years since diagnosis PD	5.7 ± *3.3*
Hoehn and Yahr stage (2/2.5/3)	4/5/3
Walking aid in daily life (yes/no)	3/9
Number of falls in previous 6 months (0/1/2/7)	5/2/4/1
Education (possible range 1–7)^a^	5.7 ± *1.3*
MMSE (possible range 0–30)^b^	27.4 ± *2.0*
LAPAQ (kcals/day)	630.3 ± *712*.1
FES-I (possible range 16–64)^c^	29.3 ± 8.2
Number of different medications used	5.3 ± 2.7

Values are mean ± standard deviation or frequency

*PD* Parkinson’s disease, *MMSE* Mini-Mental State Examination, *LAPAQ* Longitudinal Aging Study Amsterdam Physical Activity Questionnaire, *FES-I* Fall Efficacy Scale International

^a^According to Verhage and colleagues [30]

^b^Higher scores indicating better global cognitive functioning

^c^Higher scores indicating more fear of falling

### Feasibility analysis

Two of the initial 14 participants were unable to complete the whole protocol (see above). The 12 participants included in the study reported that it was doable to complete the full protocol (median Likert-scale score 1, range 1 (very easily doable) to 3 (neutral)). On average, the participants scored 10.6 ± 1.6 on the Borg-RPE, corresponding to fairly light effort. Concerning the two Portalite devices placed on their forehead, participants reported that these did not burden them during walking (median Likert-scale score 1, range 1 (no burden at all) to 2 (no burden)).

### Prefrontal cortical activity

#### PFC activity (group level)

Figure 1 shows the group averaged time course of O_2_Hb and HHb concentrations in bilateral PFC during the three dual walking tasks. In all tasks, the average O_2_Hb concentrations increased from resting conditions after starting the task and decreased again during rest after task performance. HHb concentrations remained relatively stable or showed slight reductions during task performance when compared to rest. Thus, in all three dual walking tasks, bilateral PFC activity patterns were found on a group level.

Mean O_2_Hb and HHb concentrations in left and right PFC during the three dual walking tasks are shown in Table 2 (also see Additional file 1: Figure S1). During walking while serially subtracting and walking while reciting digit spans, mean O_2_Hb concentrations in left and right PFC were significantly higher than those during rest (lower limit 95 % CI >0). Mean HHb concentrations were similar between walking tasks and rest (95 % CI includes 0). No significant differences between tasks were seen in mean O_2_Hb or HHb concentrations in left or right PFC. Effect sizes of the concentration differences between dual walking tasks were small (Table 3).

**Table 2:** Concentrations of O_2_Hb and HHb and gait performance measures during all three walking tasks

	Walking while counting	Walking while serially subtracting	Walking while reciting digit spans
fNIRS			
O_2_Hb left (μmol/L)	0.32 (−0.18–0.82)	0.49 (0.14–0.84)	0.44 (0.09–0.78)
O_2_Hb right (μmol/L)	0.31 (−0.12–0.75)	0.46 (0.12–0.81)	0.36 (0.03–0.70)
HHb left (μmol/L)	0.03 (−0.28–0.33)	0.01 (−0.15–0.17)	−0.03 (−0.16–0.10)
HHb right (μmol/L)	0.00 (−0.28–0.28)	−0.02 (−0.25–0.21)	−0.10 (−0.24–0.05)
Gait performance			
Gait speed (cm/s)	83.7 (73.7–93.7)	82.3 (71.8–92.9)	79.9 (68.2–91.6)
Cadence (steps/min)	94.6 (86.2–103)	93.2 (84.6–101.9)	94.0 (84.0–104.0)
Stride length (cm)	106.3 (97.7–114.9)	106 (96.6–115.4)	101.2 (91.5–111.0)**
Stride length variability (%)	4.2 (3.2–5.2)	4.5 (2.9–6.2)	5.4 (4.4–6.5)*
Stride time (s)	1.3 (1.2–1.4)	1.3 (1.2–1.4)	1.3 (1.2–1.5)
Stride time variability (%)	4.2 (2.4–5.9)	4.0 (2.9–5.2)	3.6 (2.7–4.6)

Values are mean (95 % confidence intervals)

*P < .05 compared to walking while counting

**P < .05 compared to both walking while talking and serially subtracting

**Table 3:** Effect sizes (Cohen’s *d*_z_) between the walking tasks for both concentrations of O2Hb and HHb and gait performance

	Walking while serially subtracting > counting	Walking while reciting digit spans > counting	Walking while serially subtracting > reciting digit spans
fNIRS			
O_2_Hb left	.29	.08	.36
O_2_Hb right	.31	.20	.27
HHb left	−.13	−.36	.43
HHb right	−.07	−.18	.42
Gait performance			
Gait speed (m/s)	−.22	−.**72**	.40
Cadence (steps/min)	−.33	−.15	−.23
Stride length (m)	−.09	−.**93**	.**73**
Stride length variability (%)	.11	**1.04**	−.32
Stride time (s)	.30	.16	.18
Stride time variability (%)	−.05	−.20	.24

Effect sizes larger than 0.5 (medium effect) printed in bold

#### PFC activity (individual level)

The O_2_Hb and HHb signals were stable over the five trials within participants (see Additional file 1: Figures S3-S7), which indicates that the wireless fNIRS system reliably measures PFC activity within subjects. However, we found large variability between participants (see standard errors in Fig. 1 and confidence intervals in Table 2). Therefore, we explored differences in individual fNIRS signal patterns and were able to identify five subgroups of participants based on signal patterns. The authors FN and JC both independently inspected the figures of each subject (individual trials and average), which resulted in two identical group distributions. Five participants had signals as hypothesized (classic task related hemodynamic response signal), an increase in O_2_Hb, and slight decrease or stable HHb during task performance (see Additional file 1: Figure S3). One participant showed an increased O_2_Hb with stable HHb, but only at the start of task performance (Additional file 1: Figure S4). Three participants were identified as non-responders, where both O_2_Hb and HHb remained relatively stable during task performance and for all task repetitions (Additional file 1: Figure S5). Two participants showed an inverse response during walking while counting; O_2_Hb decreased, while HHb remained relatively stable or decreased slightly (Additional file 1: Figure S6). During walking while serially subtracting and walking while reciting digit spans, these two participants showed no inverse response but slight increases in or stable O_2_Hb concentrations. Finally, one participant showed a highly unexpected response, with an initial increase in O_2_Hb and increase in HHb in the second half of task performance (Additional file 1: Figure S7). These subgroups could not be identified based on participant characteristics or task performance data, in which no obvious differences between subgroups were present.

### Behavioral performance

Gait performance during the dual walking tasks is shown in Table 2 (also see Additional file 1: Figure S2). For gait speed, cadence, stride time, and stride time variability, no significant differences in gait performance between any of the dual walking tasks were found. During walking while reciting digit spans, participants showed significantly decreased stride length when compared to walking while counting (*z* = −2.7, *P* = .006) and serially subtracting (*z* = −2.1, *P* = .034) and increased stride length variability when compared to walking while counting (*z* = −2.6, *P* = .010). In addition to these significant differences, for which effect sizes were medium to large (Table 3), a medium effect size (Cohen’s *d*_*z*_ = 0.72) was found for gait speed during walking while counting versus reciting digit spans. All other effect sizes were small (Table 3).

During walking while serially subtracting, participants gave an average of 25.3 (95 % CI 19.7–30.8) answers, of which on average, 98.4 % (95 % CI 97.5–99.3) was correct. The mean length of digit spans to be repeated during walking while reciting digit spans was 4.9 digits (95 % CI 4.0–5.8). Two participants were unable to perform the predetermined length of digit spans during walking; for them, the number of digits was reduced until they were able to perform (from 6 to 5 and from 7 to 4 digits). During walking while reciting digit spans, participants recited an average of 6.0 (95 % CI 5.2–6.8) spans, with a mean success rate of 88.4 % (95 % CI 79.6–97.3).

## Discussion

In this pilot study, we aimed to examine the feasibility of measuring PFC activity during dual task walking in patients with PD with use of a portable fNIRS device. Good feasibility of the portable fNIRS device was demonstrated by the fact that most participants experienced a low burden of the two fNIRS devices placed on the forehead during walking, were able to perform the different dual task walking paradigms, and reported that it took them little effort to complete the full protocol. Importantly, when averaged over all participants, fNIRS showed typical cortical activity patterns of increased oxygenated (O_2_Hb) and stable deoxygenated hemoglobin (HHb) concentrations during the three dual walking tasks when compared to rest. This further supports feasibility of using portable fNIRS to measure PFC activity during dual task walking in PD.

These findings are in line with previous studies in which fNIRS was successfully used to measure PFC activity during dual task walking in healthy young adults [24–26] and elderly [22, 23, 54]. In contrast to these studies that found differences in PFC activity levels between walking tasks, effect sizes in our study were small. Although we were able to measure PFC activity during dual task walking, future protocols need to be improved to enable detection of differences between tasks. This can possibly be done by either reducing variability in concentration measures (i.e., reducing noise), especially between participants, or choosing different tasks. Below, we will elaborate on these issues and propose protocol improvements for future work.

Large variability occurred mainly between participants, while time courses of O_2_Hb and HHb were highly stable within participants. The first potential cause of such inter-individual differences is the fNIRS optode placement on the forehead. Although the placement procedure was identical for all participants, it was based on relative distances from external landmarks (nasion and inion). It is possible that this method resulted in the targeting of slightly different brain areas due to morphological differences between subjects [19, 55]. To avoid targeting different brain areas, it could be beneficial to relocate the devices until task-related cortical activity is seen before starting measurements. The window in which to move the devices should however be limited, so that after relocation, still the region of interest is likely targeted. Also, the typically expected response of the targeted brain area should be very well known and distinct from directly surrounding brain areas [19]. Another solution could be to use portable multichannel fNIRS systems. When available, such a system could cover the whole PFC, ensuring that the target area is within the field of view [56, 57]. Another option is to use structural magnetic resonance imaging or transcranial magnetic stimulation before placing fNIRS probes to enable more precise placement over a target area, although these are costly and laborious solutions [19]. A second cause of individual differences can be systemic task responses (see also our discussion on the study limitations) such as changes in blood pressure or heart rate [58]. Adding continuous blood pressure and heart rate monitoring can help to determine the role of systemic responses in individual differences.

Future studies should try to limit individual differences by optimizing protocols as suggested above. When still present, causes of individual differences should be further investigated and taken into account when determining sample sizes and when interpreting group averaged data. Only in the case of obvious measurement errors should a participant who shows non-hypothesized fNIRS signals be excluded from analyses. For example, when it can reasonably be assumed that the wrong cortical area was targeted, when the data contains too much noise due to movement artifacts, or when systemic changes in blood pressure and subsequent changes in cerebral perfusion affect the hemodynamic response to activity, there are objective arguments to exclude a subject.

Apart from inter-individual differences, the choice of walking tasks is an important topic for improvement of future studies. First, although simple counting during walking offers a controlled condition and prevents that participants’ thoughts start to wander (“daydreaming”), it might be a rather difficult dual task itself. Accordingly, in healthy young participants walking while counting led to increased PFC activity when compared to usual walking [24]. The dual tasks we used might thus not have differed much in terms of difficulty, which would explain the lack of differences in PFC activity levels between tasks. This is further supported by the similar gait performance we saw between walking while counting and serially subtracting. And, although we did not measure it as a single task, good performances on both serially subtracting and reciting digit spans while walking suggest that these tasks might not have been much more difficult than walking while counting. Future studies should use tasks that differ more in terms of difficulty, such as usual walking without any secondary task. With usual walking as a reference task, previous studies found relatively increased PFC activity during dual tasks like walking while talking [22, 23], serially subtracting [24–26], and balancing a ping pong ball on a card [29]. A second consideration regarding the choice of task is the use of walking while reciting digit spans. Effect sizes of the difference with walking while counting for gait speed, stride length, and stride length variability were moderate to large. However, defining the number of digits to be recited was not straightforward as reflected by the two participants who were unable to perform the predefined length while walking. Also, citing the numbers seemed to provide participants with an external rhythm on which they paced their walking. Finally, walking while serially subtracting showed larger differences (effect sizes) with walking while counting for O_2_Hb concentration change. Using walking while reciting digit spans in future fNIRS dual task work is thus not recommended.

A limitation of the present study was the small sample size. Although the sample size was sufficient to fulfill our primary aims regarding feasibility, it might have been too low to find differences between dual walking tasks. Based on the variability and mean difference in O_2_Hb concentration between walking while counting and walking while serially subtracting in the present study, 85 participants would be needed to find significant differences in O_2_Hb concentrations between these tasks (power = 0.80, alpha = 0.05, two-tailed testing). However, improving protocols as suggested, to reduce variability and increase contrasts between tasks, will reduce this required sample size. Indeed, our recently published work shows that groups of 68 PD patients and 38 healthy controls were sufficient to find O_2_Hb concentration differences between complex walking tasks within groups [13].

Also, we cannot generalize findings to the broader PD population. Although all participants were identified with an increased risk of falling, they were able to walk at least 5 min unassisted, their cognitive functioning was relatively well, and all were in mild to moderate stages of PD. Two patients who had passed screening for inclusion and exclusion criteria were unable to complete the protocol. Although no large differences were present, these two participants were slightly older and had a longer history of PD than the mean of those participants who were able to complete the protocol. This raises doubt whether the protocol is feasible for more frail and severely affected PD patients, which is a subject for future research.

Other limitations concern our use of fNIRS. We did not control for superficial (e.g., skin) hemodynamics [59, 60], for example, by using a short reference channel [59]. Thus, we cannot rule out the influence of blood flow through skin or the occipitofrontalis muscle, which might have increased during task performance [61]. However, in frontal brain areas, high correlations were found between fNIRS signals and fMRI signals from the cortical gray matter layer while performing several cognitive tasks [62, 63]. These correlations were higher than correlations between fNIRS signals and soft tissue fMRI signals [62]. Although our fNIRS signals are likely affected by skin blood flow, we can still assume based on these data that they do reflect cortical neuronal activity. A further limitation is that only PFC activity levels were measured. We did not measure any other cortical areas to ensure specificity of signals to PFC instead of whole brain effects. The use of a multiple channel fNIRS system would allow for measurements of multiple cortical areas and thereby to differentiate between region-specific and global effects; however, the increase in weight of such a system may reduce the feasibility. Besides cognitive tasks, postural changes and walking exercise may all lead to changes in blood pressure which in turn can affect global cerebral blood flow [18]. For this reason, it is advisable to measure blood pressure simultaneously with fNIRS.

## Conclusions

In this study, we showed that using two small, lightweight, portable fNIRS devices placed on the forehead is feasible to measure PFC activity during dual task walking in PD patients. We provided recommendations for improvements of protocols to increase the sensitivity to detect differences in PFC activity and behavioral performance between dual walking tasks and potentially between populations (PD patients vs. healthy age-matched controls) and/or changes over time (neurorehabilitation training effects). With improved protocols, portable fNIRS seems to be a very promising tool to further study the role of the PFC in mechanisms underlying difficulties in dual task walking in PD.

## Additional file

Additional file 1: Supplementary figures can be found in the document “Additional file 1—Supplementary figures.pdf” (PDF 1324 kb)

## Abbreviations

CV: Coefficient of variability
DPF: Differential path length factor
EEG: Electroencephalography
FES-I: Fall Efficacy Scale International
fMRI: Functional magnetic resonance imaging
fNIRS: Functional near-infrared spectroscopy
Hb: Hemoglobin
HHb: Deoxygenated hemoglobin
LAPAQ: Longitudinal Aging Study Amsterdam Physical Activity Questionnaire
MARA: Movement artifact reduction algorithm
MMSE: Mini-Mental State Examination
O_2_Hb: Oxygenated hemoglobin
PD: Parkinson’s disease
PFC: Prefrontal cortex
RPE: Rating of perceived exertion
WAIS: Wechsler Adult Intelligence Scale
