# Supplementary material for: Measuring prefrontal cortical activity during dual task walking in patients with Parkinson’s disease: feasibility of using a new portable fNIRS device
Source: Pilot Feasibility Stud. 2016 Sep 23;2:59. doi: 10.1186/s40814-016-0099-2 (PMC5154104; doi:10.1186/s40814-016-0099-2)
Supplement: Additional file 1: — Supplementary figures can be found in the document “Additional file 1—Supplementary figures.pdf” (PDF 1324 kb) [file 40814_2016_99_MOESM1_ESM.pdf]

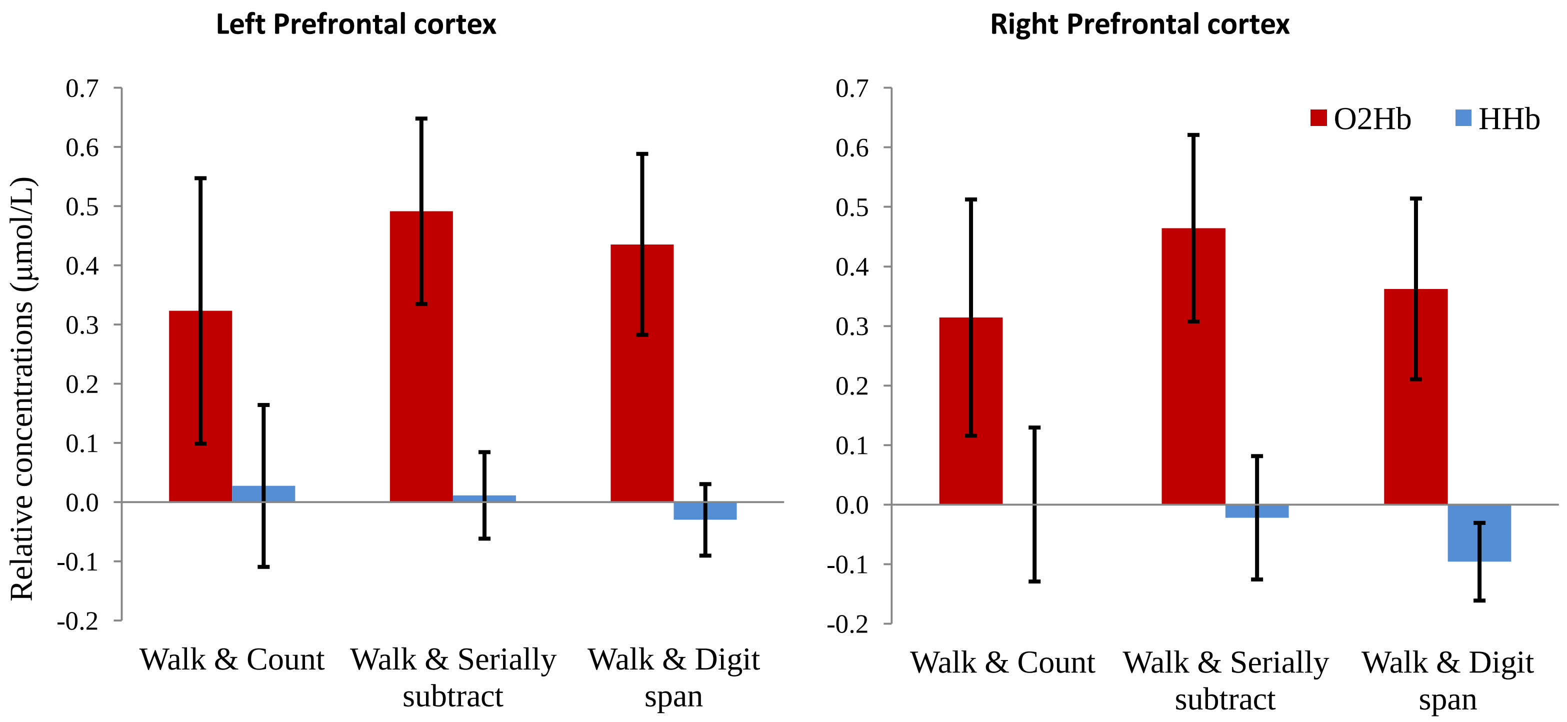

Supplementary figure 1 – Mean concentrations of O2Hb (red) and HHb (blue) relative to rest (µmol/L) in left and right prefrontal cortices during walk & count, walk & serially subtract and walk & digit span, mean ± sem.

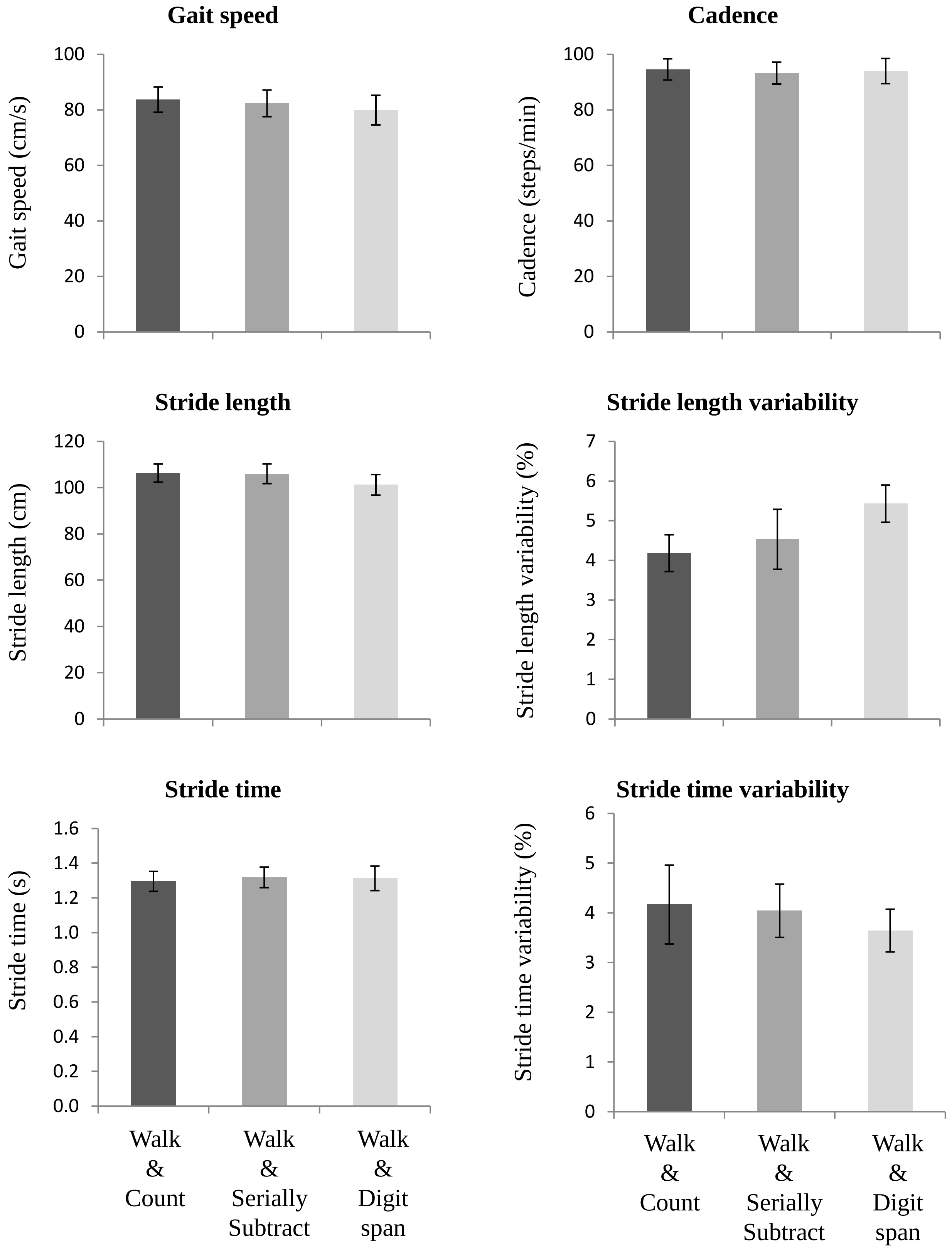

Supplementary figure 2 – Gait performance measures during walk & count, walk & serially subtract and walk & digit span, mean ± sem.

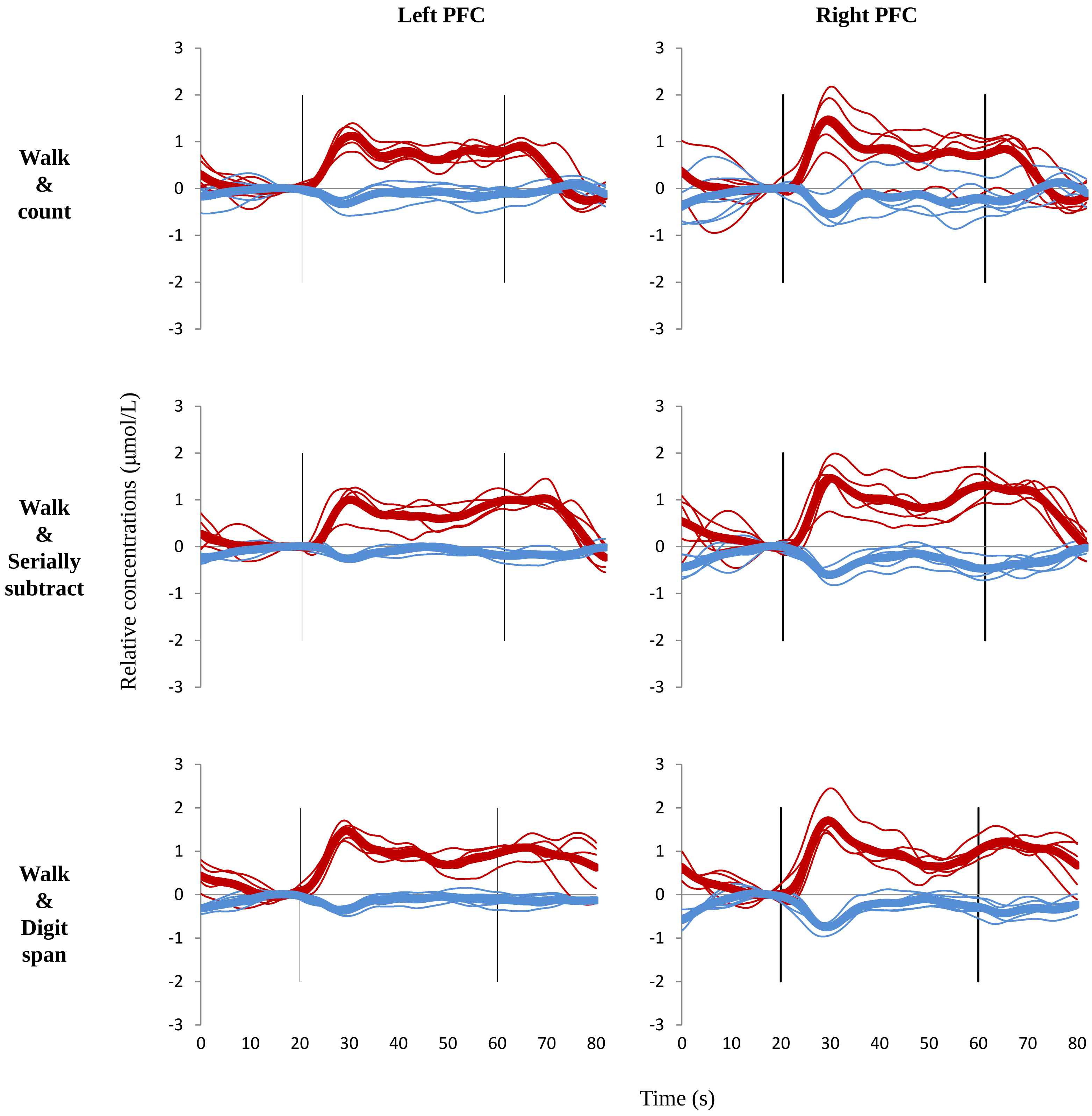

Supplementary figure 3 – Average (thick line) and individual trial (thin lines) time courses of oxygenated hemoglobin (O2Hb, red) and deoxygenated hemoglobin (HHb, blue) of a representative participant showing a timecourse as hypothesized. Vertical black lines indicate start and end of task performance. PFC = prefrontal cortex

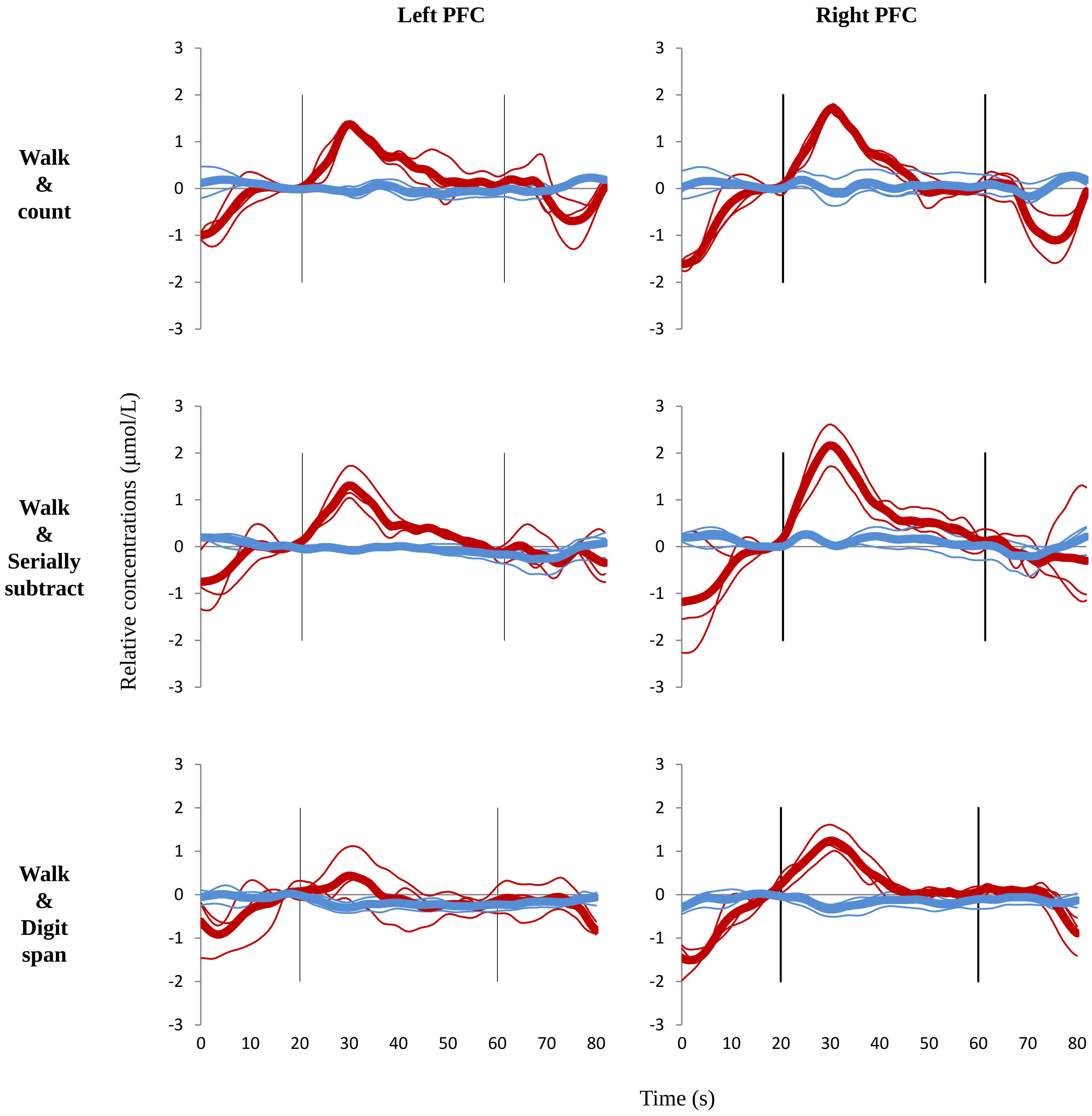

Supplementary figure 4 – Average (thick line) and individual trial (thin lines) time courses of oxygenated hemoglobin (O2Hb, red) and deoxygenated hemoglobin (HHb, blue) of the participant showing an initial activation pattern. Vertical black lines indicate start and end of task performance. PFC = prefrontal cortex

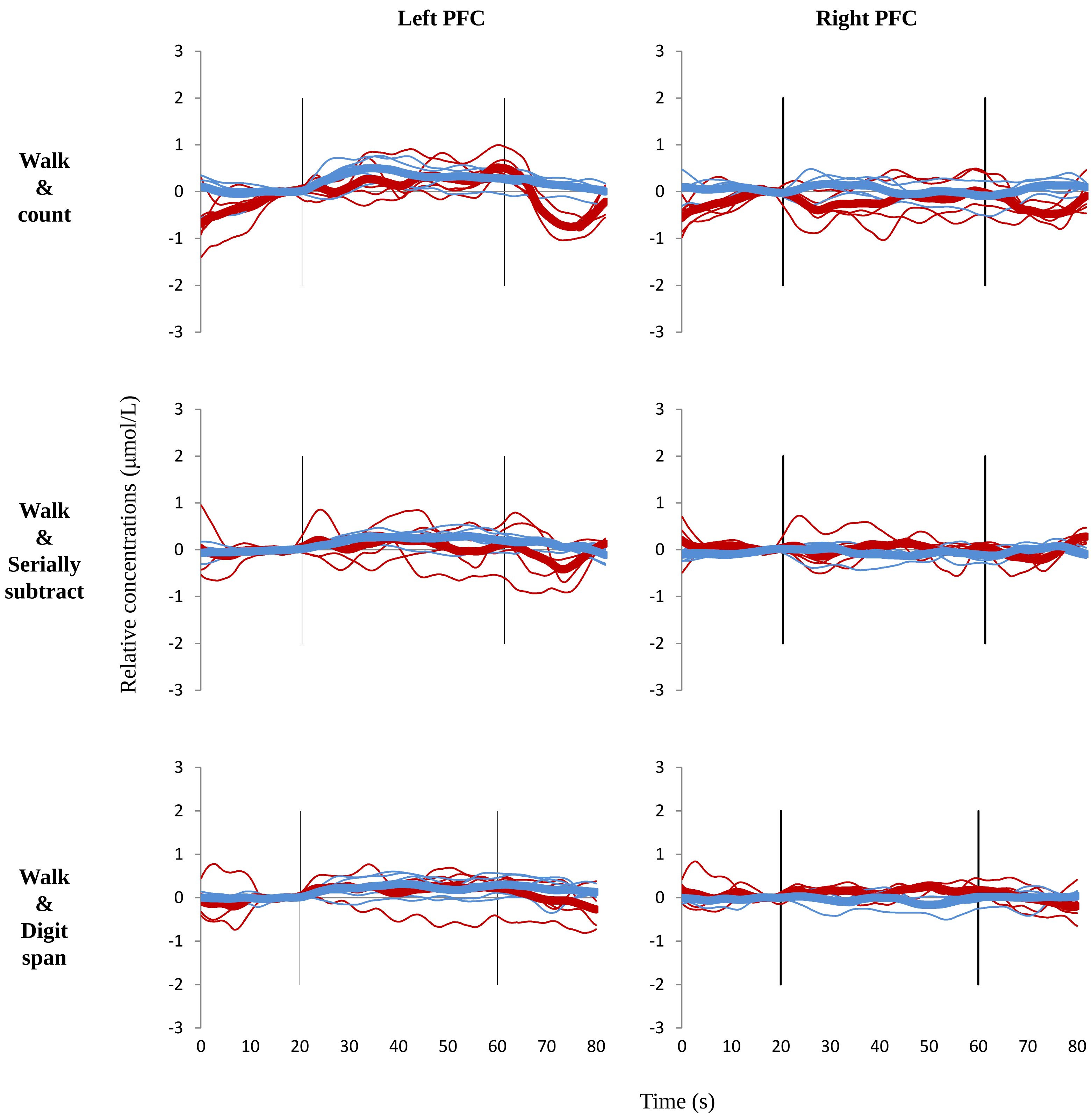

Supplementary figure 5 – Average (thick line) and individual trial (thin lines) time courses of oxygenated hemoglobin (O2Hb, red) and deoxygenated hemoglobin (HHb, blue) of a representative non-responder. Vertical black lines indicate start and end of task performance. PFC = prefrontal cortex

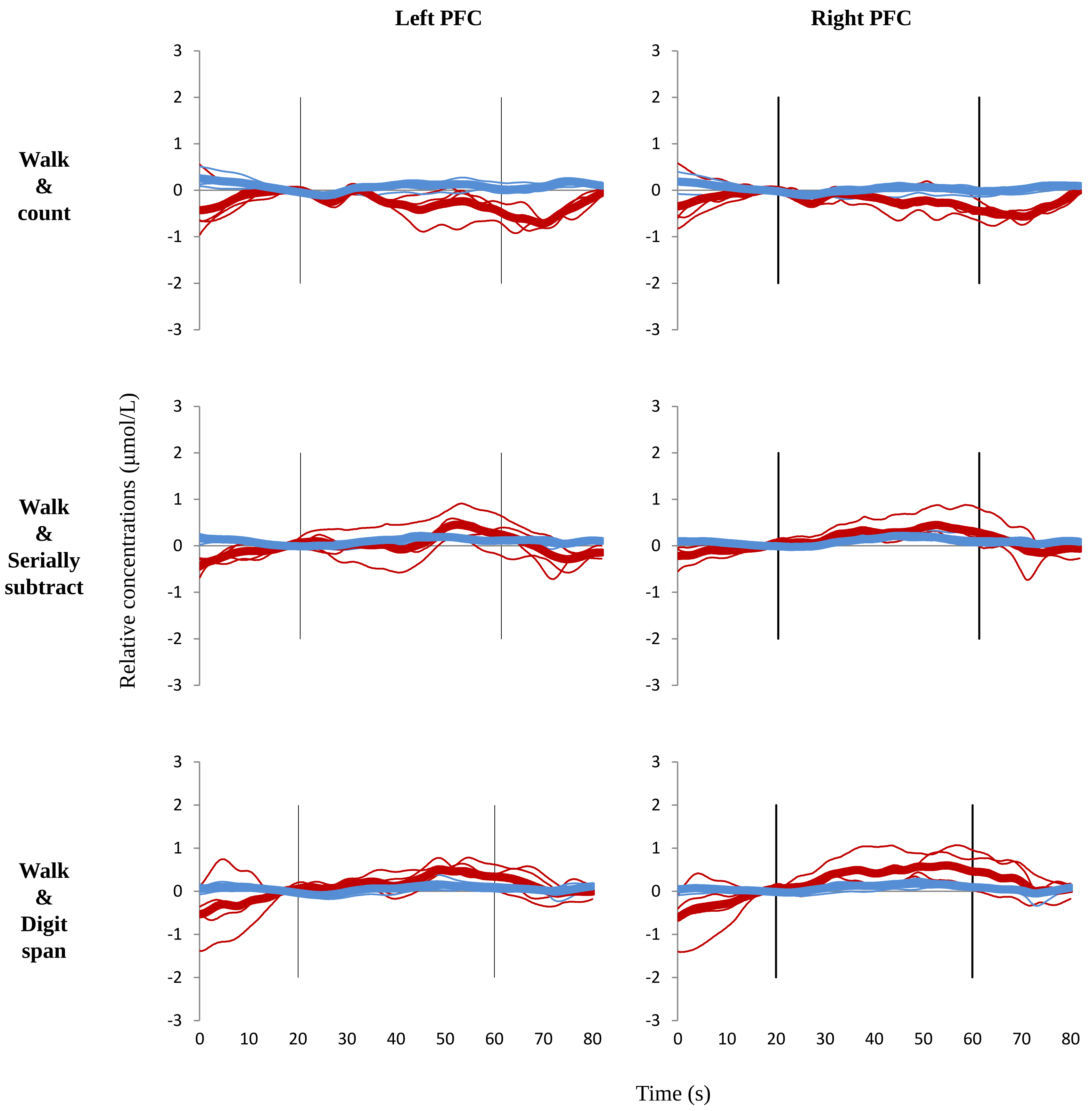

Supplementary figure 6 – Average (thick line) and individual trial (thin lines) time courses of oxygenated hemoglobin (O2Hb, red) and deoxygenated hemoglobin (HHb, blue) of a representative participant showing reduced activation during walking while counting. Vertical black lines indicate start and end of task performance. PFC = prefrontal cortex

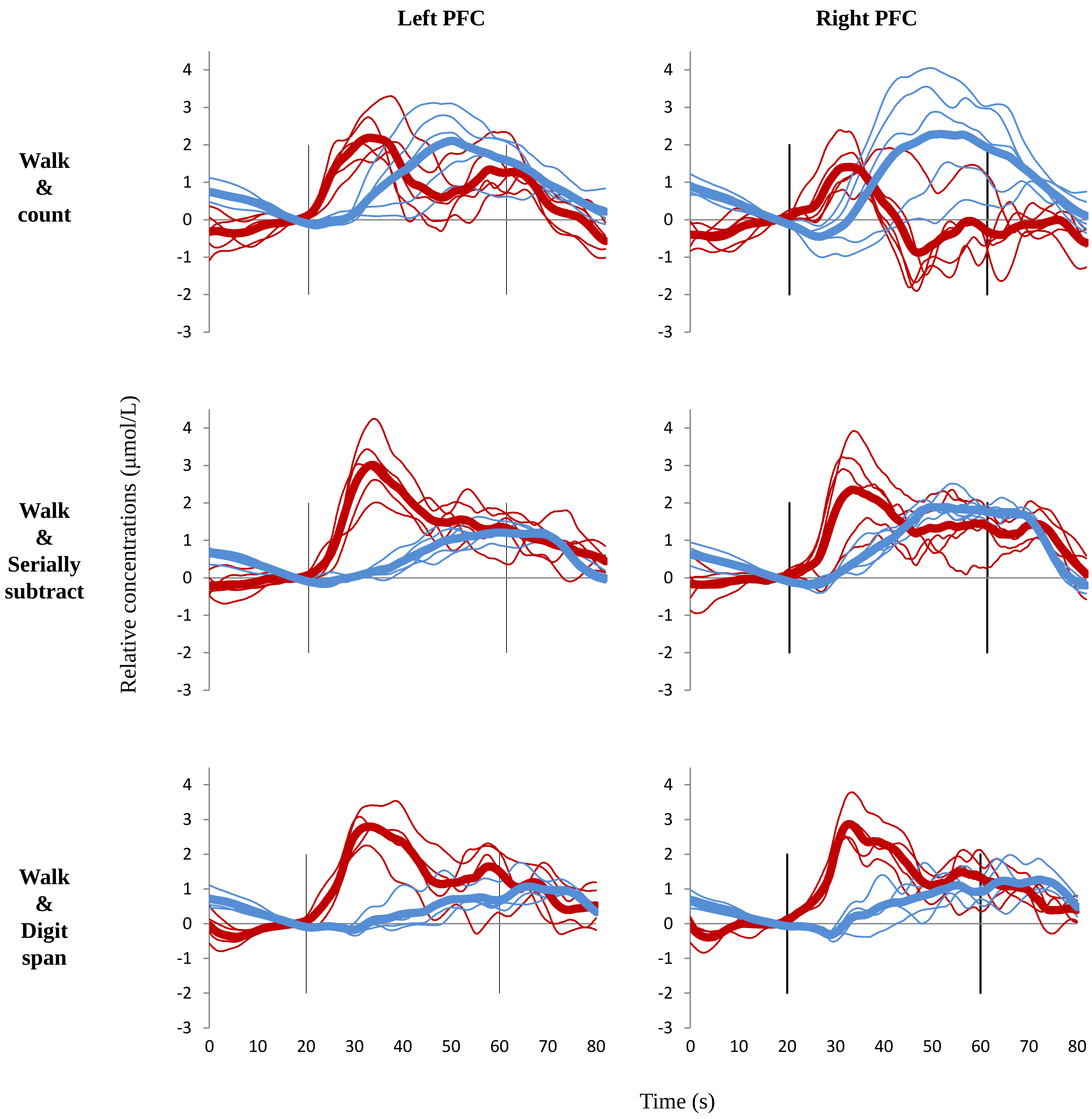

Supplementary figure 7 – Average (thick line) and individual trial (thin lines) time courses of oxygenated hemoglobin (O2Hb, red) and deoxygenated hemoglobin (HHb, blue) of the participant showing an unexpected pattern. Vertical black lines indicate start and end of task performance. PFC = prefrontal cortex
